# Haptic shape discrimination and interhemispheric communication

**DOI:** 10.1038/s41598-017-18691-2

**Published:** 2018-01-10

**Authors:** Catherine J. Dowell, J. Farley Norman, Jackie R. Moment, Lindsey M. Shain, Hideko F. Norman, Flip Phillips, Astrid M. L. Kappers

**Affiliations:** 1 Western Kentucky University, Department of Psychological Sciences, Ogden College of Science and Engineering, Bowling Green, Kentucky 42101-2030 USA; 2 Skidmore College, Department of Psychology & Neuroscience Program, Saratoga Springs, New York 12866 USA; 3 Vrije Universiteit, Department of Human Movement Sciences, MOVE Research Institute, 1081 HV Amsterdam, Netherlands

## Abstract

In three experiments participants haptically discriminated object shape using unimanual (single hand explored two objects) and bimanual exploration (both hands were used, but each hand, left or right, explored a separate object). Such haptic exploration (one versus two hands) requires somatosensory processing in either only one or both cerebral hemispheres; previous studies related to the perception of shape/curvature found superior performance for unimanual exploration, indicating that shape comparison is more effective when only one hemisphere is utilized. The current results, obtained for naturally shaped solid objects (bell peppers, *Capsicum annuum*) and simple cylindrical surfaces demonstrate otherwise: bimanual haptic exploration can be as effective as unimanual exploration, showing that there is no necessary reduction in ability when haptic shape comparison requires interhemispheric communication. We found that while successive bimanual exploration produced high shape discriminability, the participants’ bimanual performance deteriorated for simultaneous shape comparisons. This outcome suggests that either interhemispheric interference or the need to attend to multiple objects simultaneously reduces shape discrimination ability. The current results also reveal a significant effect of age: older adults’ shape discrimination abilities are moderately reduced relative to younger adults, regardless of how objects are manipulated (left hand only, right hand only, or bimanual exploration).

## Introduction

We humans explore and manipulate environmental objects primarily with our hands. Multiple studies over the past half century^1–5^ have demonstrated that for some tactile and proprioceptive tasks, performance for two hands is as good, or better, than that obtained from the usage of a single hand. Lappin and Foulke^1^, for example, required participants to detect and then count single-dot raised bumps mixed in with two-dot patterns of bumps. Their participants’ performance was faster and more accurate when two fingers on separate hands were used to scan the stimuli than for unimanual conditions where two fingers on a single hand were employed. Similarly, in an investigation evaluating the discrimination of object stiffness/softness, Plaisier and Ernst^2^ found that their participants’ judgments were most precise when two hands were used for haptic exploration.

Tactual sensory information accompanying the haptic exploration of object surfaces eventually reaches anterior parietal cortex. A hierarchical organization exists within somatosensory cortex such that tactile information initially arrives at area 3b, then spreads to areas 1 and 2^6–9^. Eventually, information about touch arrives in posterior parietal cortex (e.g., area 5)^10^. Receptive field properties change substantially along this pathway: in area 3b, neurons subserving the hand receive excitatory input exclusively from the contralateral hand and fingers^9^ (there is ipsilateral input, but its’ function is to suppress and modulate neuronal activity arising from input from the contralateral hand^11,12^). While neurons in 3b are only excited by tactile input from the contralateral hand, neurons in areas 2 and 5 have bilateral receptive fields and will thus respond to appropriate tactile input from both hands^6,13,14^. This bilateral functionality depends upon interhemispheric communication through the corpus callosum; if the postcentral gyrus in one cerebral hemisphere is destroyed or ablated, the sensitivity of somatosensory neurons in the other hemisphere’s area 2 or 5 to ipsilateral tactile information is eliminated^15^. The importance of the corpus callosum for the human sense of touch was behaviorally demonstrated by Gazzaniga, Bogen, and Sperry^16^. These researchers studied the tactile abilities of a man whose cerebral hemispheres had been disconnected as a treatment for severe epilepsy. After being blindfolded, the patient was instructed to point to locations where he had been touched. Correct localizations only occurred when both the area of skin touched and the hand used to point were located on the same side of the body. Gazzaniga *et al*. concluded (p. 213) “that somatosensory information from each half of the body below the neck is projected in this patient only to the contralateral hemisphere, and further, that the sensations involved are not accessible in any direct way to the ipsilateral hemisphere”.

As we have seen, bilateral sensitivity to touch requires the interhemispheric transfer of sensory information across the corpus callosum^8,12,15^. Haptic comparison of objects using two separate hands, therefore, necessitates cooperation between the two cerebral hemispheres. In contrast, object comparison with a single hand only requires somatosensory processing within a single hemisphere. Multiple previous studies investigating the perception of object shape and structure^17–19^ have demonstrated that bimanual haptic performance is lower than unimanual performance (an additional study, however, found no overall difference in performance between the usage of one and two hands^20^). In all of these previous studies, relatively simple objects were haptically explored by participants (cylindrical curvatures, quadric surfaces, tactile gratings, & aggregates of cubes). No study has yet investigated whether the previously reported advantage for unimanual haptic exploration (and corresponding disadvantage for bimanual exploration) occurs for more ecologically valid objects (i.e., those that possess more naturalistic and complex solid shapes). One purpose of the current study is to remedy this deficit–at the moment, we do not know the extent to which the two cerebral hemispheres can cooperate in order to bimanually perceive and discriminate natural solid shape. At this point, it is important to note that psychophysical findings obtained for simple geometric objects do not necessarily generalize to objects that possess more complex and/or naturalistic geometrical structure. For example, Norman *et al*.^21^ found considerable viewpoint invariance for shape discrimination tasks involving naturally-shaped bell peppers, but very little viewpoint invariance was obtained^22^ for sinusoidally-modulated spheres. Another current unknown is whether aging differentially affects the uni- and bimanual perception of object shape. Previous research has demonstrated that aging is associated with reduced interhemispheric communication^23–28^. Because of this age-related neurophysiological deterioration in hemispheric connectivity, one would expect that older adults’ decrement in performance (accompanying bimanual exploration) would be even larger than whatever decrement occurs for younger adults. A second purpose of the current study was to evaluate this hypothesis.

## Experiment 1

### Method

#### Apparatus

An Apple PowerMacintosh G4 computer was used to randomly order the presentation of the experimental stimuli.

#### Experimental Stimuli

**Figure 1:**
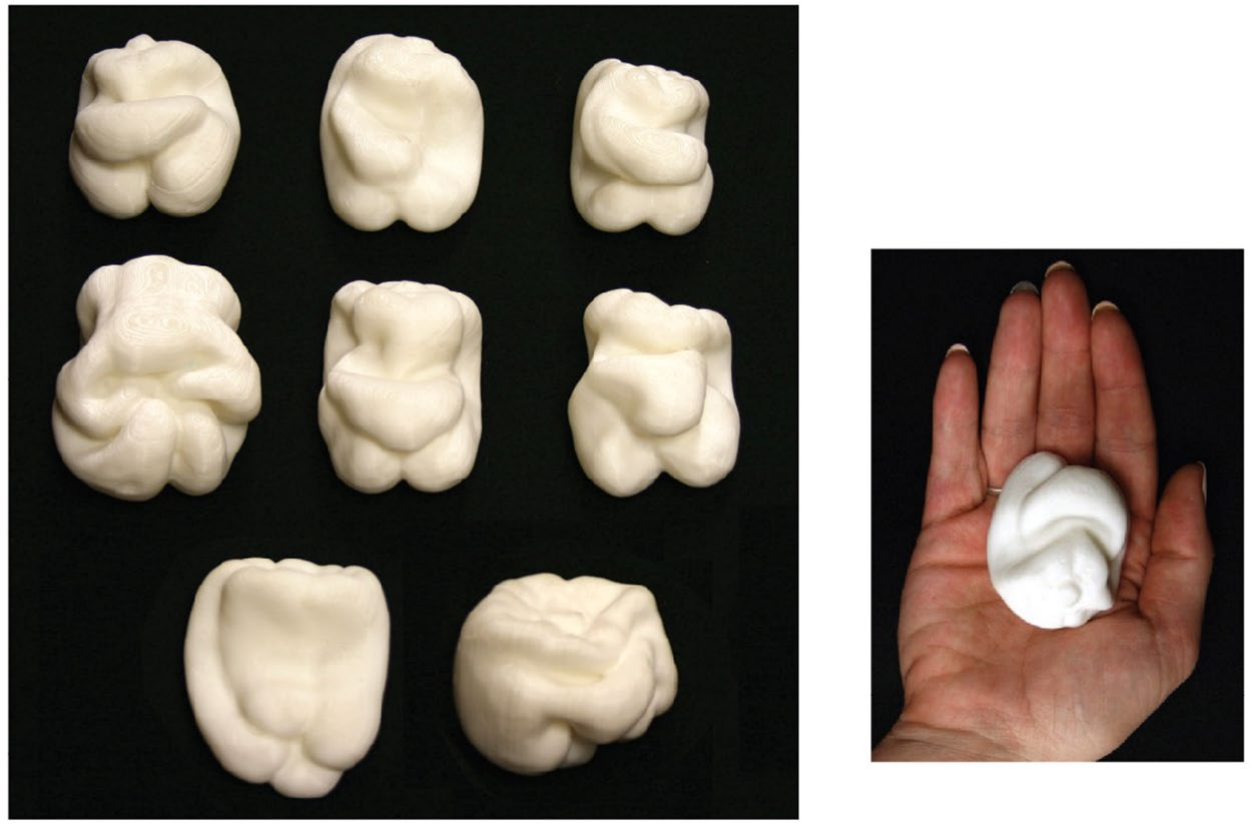
The left panel shows photographs of the eight stimulus objects (scaled replicas of bell peppers, *Capsicum annuum*) used in Experiment 1. Progressing from upper left to bottom right are objects 1, 2, 3, 5, 7, 8, 11, and 12. The right panel shows object 1 in the palm of a participant’s hand; the objects used in this experiment were scaled to one-eighth of the volume of the original bell pepper replicas that were created by Norman *et al*.^31^.

The stimulus objects were 8 of the 12 plastic replicas of naturally-shaped bell peppers (*Capsicum annuum*) that have been used in previous research^21,29–31^. In order to permit haptic exploration by a single hand, the large original-sized bell peppers (average volume was 350 cm^3^) were reduced in volume to one-eighth of their original size (see Fig. 1). To do this, the original bell peppers were first laser scanned (NextEngine Laser Scanner). The x, y, and z Cartesian coordinates of the resulting 3-D models (resolution of about 74,000 triangular polygons) were scaled by one-half (scaling by a factor of 0.5 in all dimensions results in a reduction in volume to one-eighth of the original size, 0.5 × 0.5 × 0.5 = 0.125). Following this reduction in volume, two sets of the bell peppers were then printed in PLA plastic (polylactic acid) using a Bits From Bytes 3D Touch printer.

#### Procedure

The participants were required to perform a same versus different shape discrimination task. On any given trial, participants haptically explored two objects successively for three seconds each, separated by a 3-second interstimulus interval (ISI); they were then required to judge whether the objects possessed the same shape or had different shapes. These parameters (3 sec presentation, 3 sec ISI) have been successfully used in past studies^21,29–31^. The stimulus objects were randomly oriented by the experimenter for every presentation. There were three between-subjects experimental conditions where participants haptically explored the stimulus objects using either (1) their right hand only, (2) their left hand only, or (3) both hands. It is important to note that in the both hands condition, different hands were used to explore the two stimulus objects on any given trial (left hand explored only one object, while the right hand explored a completely separate object); therefore, in this condition, any given hand (left or right) haptically explored only one object per trial (our procedures were completely unlike those of Squeri *et al*.^3^; in their bimanual conditions, each hand explored both stimuli within any given trial). For those participants in the both hands condition, half judged object shape with the left hand first, while the remainder judged object shape with the right hand first. The participants never saw the stimulus objects; they were always haptically manipulated behind an occluding surface.

Each participant made a total of 96 shape judgments (48 “same” trials and 48 “different” trials). As was pointed out in an earlier section, we used a subset of the 12 bell pepper replicas created by Norman *et al*.^31^: in the current experiment, we only used objects 1, 2, 3, 5, 7, 8, 11, and 12. These individual objects were chosen, because pairs of objects formed from these 8 bell peppers (objects 1 and 3, objects 1 and 7, objects 2 and 11, objects 3 and 7, objects 3 and 8, objects 5 and 12) are especially challenging for human participants (see Table 1 of Norman *et al*.^31^). Within a block of 96 trials, there were 8 presentations of the 6 “different” pairs of objects (1 and 3, 1 and 7, 2 and 11, 3 and 7, 3 and 8, 5 and 12), resulting in a total of 48 “different trials”. In addition, there were 48 “same trials” within each block of 96 total trials where one of the 8 individual objects (randomly chosen) would be paired with itself (i.e., presented twice successively). The order of the various objects and same versus different trials was determined randomly for each individual participant.

In addition to evaluating the participants’ haptic shape discrimination ability, we assessed their manual dexterity using the Moberg Pick-up Test^32–37^; if an older participant, for example, possesses reduced manual dexterity, it could conceivably affect their ability to haptically perceive 3-D object shape. In this test, the participants pick up 12 small metal objects (one at a time) and place them within a container as rapidly as possible. Examples of the objects would include a wing nut, a wood screw, a safety pin, a key, etc. The cumulative time needed to pick up all of the objects is measured both with and without vision; good manual dexterity is associated with shorter (i.e., faster) pick-up times. Our participants completed the pick-up test twice for each hand that was used for haptic shape exploration (some participants only used their right hand; other participants only used their left hand; others used both hands); the shorter pick-up time (i.e., best performance) of the two measured was used in the resulting analyses.

#### Participants

Forty-eight younger and older adults participated in the experiment (8 participants for each of the 6 combinations of experimental condition and age group). All participants were either right handed (47) or ambidextrous (1); none were left handed. Twenty-four of the participants were older (*M* = 73.4 years of age, *SD* = 6.1, range = 62 to 87 years) and 24 were younger (*M* = 22.5 years of age, *SD* = 3.2, range = 19 to 31 years). All participants were naive regarding the purpose of the experiment. The study was approved by the Institutional Review Board of Western Kentucky University, and each participant signed an informed consent document prior to testing. Our research was carried out in accordance with the Code of Ethics of the World Medical Association (Declaration of Helsinki).

#### Data availability

The data that support the findings of this study are available from the corresponding author upon reasonable request.

### Results and Discussion

**Figure 2:**
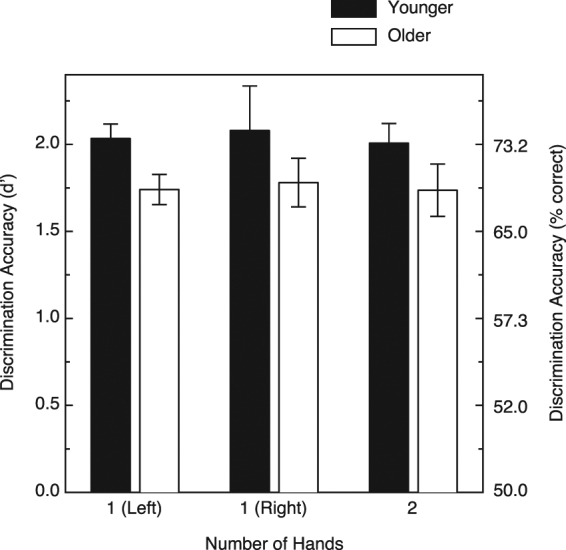
Results for Experiment 1. The younger and older participants’ shape discrimination accuracies are plotted in terms of d’ for the three unimanual and bimanual haptic exploration conditions. The error bars indicate ±1 SE. The corresponding values for percent correct were derived from Table A5.3 of Macmillan and Creelman^38^.

**Figure 3:**
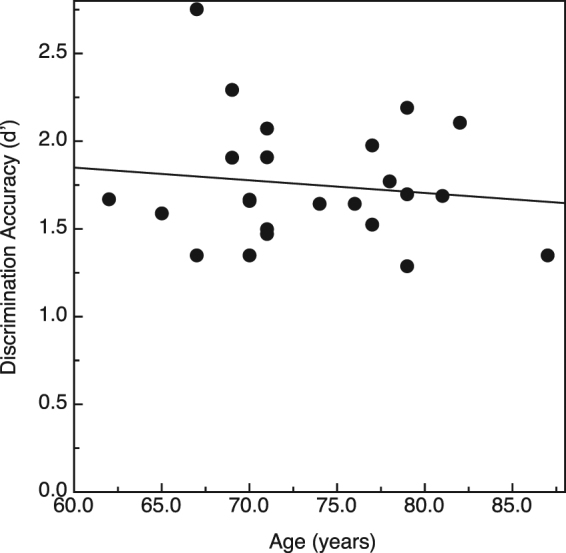
Results for Experiment 1. The older participants’ shape discrimination performances (d′) are plotted as a function of their individual ages. The solid line depicts the best-fitting linear regression.

The participants’ results are shown in Fig. 2; the figure plots shape discrimination performance in terms of d′ (the signal detection measure of perceptual sensitivity^38^) as a function of the various haptic exploration conditions. It is readily apparent from an inspection of Fig. 2 that there was no effect of the number of hands, i.e., no difference in performance either between the use of one hand and two hands or between the left-hand-only and right-hand-only conditions (F(2, 42) = 0.08, *p* = 0.92, η^2^_p_ = 0.004). While there was no effect of the various hand conditions, there was, however, a significant and adverse effect of increased age (F(1, 42) = 5.5, *p* < 0.025, η^2^_p_ = 0.12), such that the older participants’ discrimination accuracy was modestly reduced relative to that of the younger participants. The interaction between age and hand condition was not significant (F(2, 42) = 0.01, *p* = 0.995, η^2^_p_ < 0.001) reflecting the fact that there was no variation in performance across the various hand conditions for either the younger or older adults. Given that there was an overall significant effect of age upon shape discrimination performance and that the ages of our older participants varied widely (62 to 87 years), we decided to determine whether there was variation in performance within our sample of older adults. A plot of the older adults’ shape discrimination performance as a function of their individual ages is provided in Fig. 3. It is interesting that while we found an overall adverse effect of age (Fig. 2) such that the older adults performed more poorly than the younger adults, there was no deterioration of performance with increasing age (r = −0.13, p = 0.56, 2-tailed) within our sample of older participants despite the fact that the ages of our youngest older participant (62 years) and oldest older participant (87 years) differed by a quarter of a century.

**Figure 4:**
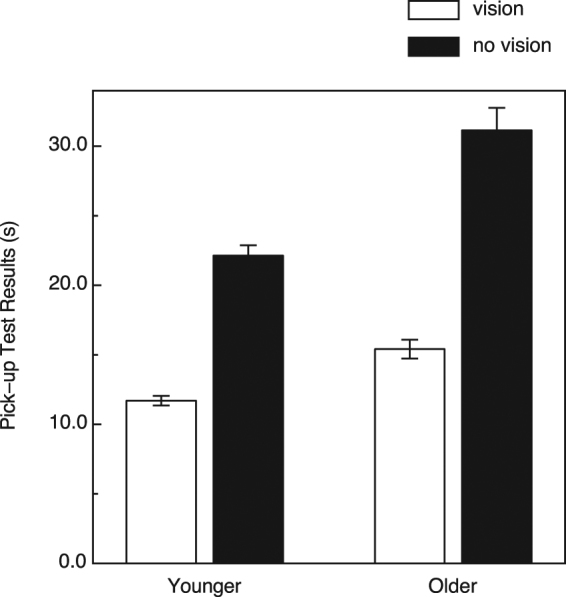
Results for Experiment 1. The younger and older participants’ performances for the pick-up test of manual dexterity are plotted for the conditions with and without vision. The error bars indicate ±1 SE.

The results of the assessment of manual dexterity (i.e., Moberg pick-up test) are shown in Fig. 4. Not surprisingly, performance was best when vision was permitted and deteriorated when only tactual sensory input was allowed (F(1, 46) = 296.8, *p* < 0.000001, η^2^_p_ = 0.87). There was also a significant main effect of age (F(1, 46) = 31.4, *p* < 0.000001, η^2^_p_ = 0.41) as well as an age × vision/no-vision interaction (F(1, 46) = 12.2, *p* = 0.001, η^2^_p_ = 0.21). It is not surprising that the older adults, on average, possessed reduced manual dexterity^36,37^. What is important is that while there were large variations in manual dexterity among our sample of older participants (e.g., reflected by without vision pick-up times), these variations in dexterity did not significantly influence (i.e., did not correlate: r = −0.277, p = 0.19, 2-tailed) their haptic shape discrimination performance; even if this relationship had been statistically significant, the older participants’ manual dexterities would have accounted for only 7.7 percent of the variance (r^2^ = 0.077) in their haptic shape discrimination performance.

## Experiment 2

The results of Experiment 1 (Fig. 2) clearly demonstrate that there was no difference in shape discrimination performance between one-handed and two-handed haptic exploration (i.e., usage of one or two cerebral hemispheres to judge shape). This outcome is quite different from a number of previous studies^17,18^ that found a superiority in performance for unimanual haptic exploration. One obvious possibility for the difference in outcome is the stimulus objects themselves: the relevant previous studies used simple curved surfaces, such as cylindrical and quadric surfaces, whereas in the current Experiment 1, participants were required to discriminate complex and naturalistic object shapes (replicas of bell peppers, *Capsicum annuum*, see Fig. 1). The purpose of Experiment 2 was to test this possibility, that the difference in object shapes themselves produced the difference in outcome. In the current Experiment 2, we followed the basic procedure of Experiment 1, but used cylindrical surfaces developed by Pont, Kappers, and Koenderink^39^.

### Method

#### Apparatus

An Apple MacBook computer was used to randomly determine the presentation order of the experimental stimuli and to collect the participants’ responses.

#### Experimental Stimuli

The stimulus objects were the same cylindrical curved blocks used by Pont *et al*.^39^: the overall dimensions were 20 × 2 × ~5 cm (length × width × height). The top of the blocks (which were haptically explored by participants using their index finger) possessed convex and concave curvatures of 0.2, 0.6, and 1.0 /m. These curved blocks were created using a computer-controlled milling machine; they are plastic (PVC, polyvinyl chloride), and their surfaces are completely smooth to the touch (i.e., no texture).

#### Procedure

The basic procedure was similar to that used in Experiment 1. The participants haptically explored the top surface of two curved blocks successively (convex & concave of the same curvature magnitude) and made a shape judgment. For these stimuli, the participants were required to judge which of the two stimulus objects on any given trial was convex, the first or the second (i.e., 2AFC temporal forced choice). As in previous research^33,40,41^ the participants were limited by an aperture to feeling the middle 10 cm portion of the curved surfaces. Each curved block was presented (and haptically explored by the participant) for 5 seconds; the successive stimulus presentations were once again separated by a 3-second ISI. The participants haptically explored each object behind an occluding surface, so that they never saw the actual experimental stimuli. Just as in Experiment 1, the objects were haptically explored using either one (right) or two hands; in the two hands condition, the left hand would feel/explore one of the stimulus surfaces on any given trial, while the right hand would feel/explore the other. In this experiment, one versus two hands was a within-subjects factor. Each participant judged surface shape with one hand and with two hands on separate days: 3 participants judged surface shape using one hand first, while the remaining 2 participants judged surface shape with two hands first.

As in previous studies^33,40^, within any given experimental session participants judged stimulus surfaces with the largest curvature first (1.0 /m) and progressed to the medium and smallest curvature magnitudes (0.6 and 0.2 /m, respectively). Each participant made a total of 40 shape judgments for each curvature magnitude; the order of the 20 convex presented first trials and 20 convex presented second trials within any particular block was completely random.

#### Participants

Five younger adults participated in the experiment (mean age = 23.4 years, sd = 1.1). All participants were naive regarding the purpose of the experiment. The study was approved by the Institutional Review Board of Western Kentucky University, and each participant signed an informed consent document prior to testing. Our research was carried out in accordance with the Code of Ethics of the World Medical Association (Declaration of Helsinki).

#### Data availability

The data that support the findings of this study are available from the corresponding author upon reasonable request.

### Results and Discussion

**Figure 5:**
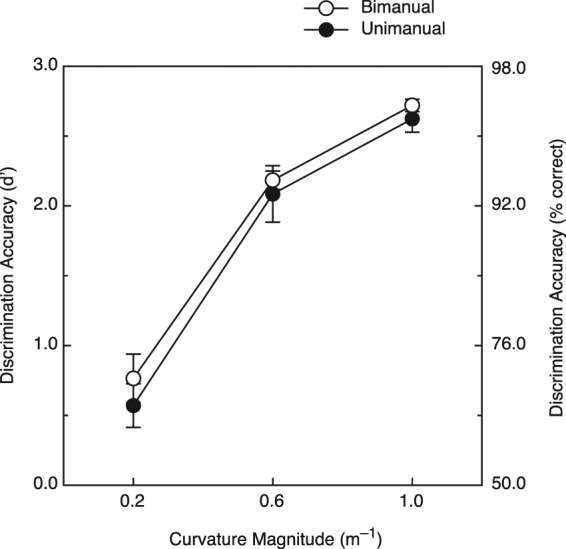
Results for Experiment 2. The participants’ shape discrimination accuracies (d′) for both haptic exploration conditions are plotted as a function of the stimulus curvature magnitude. The error bars indicate ±1 SE. The corresponding values for percent correct were derived from Table A5.2 of Macmillan and Creelman^38^.

The participants’ results are shown in Fig. 5. It is readily apparent that while there was a large effect of the curvature magnitude upon shape discrimination performance (F(2, 8) = 118.4, p < 0.000001; η^2^_p_ = 0.97), there was no effect of unimanual versus bimanual haptic exploration (F(1, 4) = 0.8, p = 0.43, η^2^_p_ = 0.159). In addition, the effect of curvature was strikingly similar for both unimanual and bimanual exploration conditions (i.e., no hand condition × curvature interaction, F(2, 8) = 0.3, p = 0.74, η^2^_p_ = 0.072).

## Experiment 3

In neither Experiment 1 nor Experiment 2 was there an effect of unimanual versus bimanual haptic exploration. This clear outcome (see Figs 2 and 5) is quite different from a number of previous studies^17,18^ that found a superiority for unimanual exploration. A comparison of the methodology of current and past experiments reveals a potential explanation for the conflicting outcomes: in the previous studies, participants in the two-handed conditions were allowed simultaneous stimulus exploration, while the comparison in the one-handed conditions was always successive. It is thus possible that the previously reported difference in shape discrimination performance between unimanual and bimanual conditions actually reflects a difference between simultaneous and successive haptic exploration. The purpose of the current experiment was to test this possibility.

### Method

#### Apparatus and Experimental Stimuli

The apparatus and experimental stimuli were the same as those used in Experiment 2.

#### Procedure

The procedures were identical to those used in the two-handed/bimanual condition of Experiment 2. The only exception was that the haptic exploration of the two stimulus objects on any given trial was simultaneous rather than successive; for example, the participants explored a convex (or concave) stimulus with the right hand and a concave (or convex) stimulus with the left hand. On any given trial, the participants’ task was to judge whether the convex stimulus was located left or right (2AFC spatial forced choice).

#### Participants

The same 5 younger adults who had made successive judgments in Experiment 2 participated in the current experiment. All participants were once again naive regarding the purpose of the experiment. The study was approved by the Institutional Review Board of Western Kentucky University, and each participant signed an informed consent document prior to testing. Our research was carried out in accordance with the Code of Ethics of the World Medical Association (Declaration of Helsinki).

#### Data availability

The data that support the findings of this study are available from the corresponding author upon reasonable request.

### Results and Discussion

**Figure 6:**
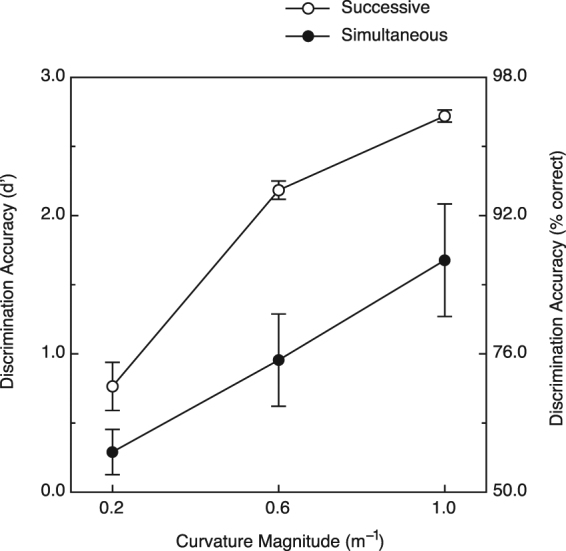
Experimental results. The participants’ shape discrimination accuracies (d′) for Experiment 3 (simultaneous stimulus exploration) are plotted as a function of the stimulus curvature magnitude. The analogous performance for Experiment 2 (successive stimulus exploration) is plotted for comparison. The error bars indicate ±1 SE. The corresponding values for percent correct were derived from Table A5.2 of Macmillan and Creelman^38^.

The results obtained for simultaneous haptic exploration are shown in Fig. 6 (filled circles) along with the participants’ successive exploration results from Experiment 2 (open circles). It is clear that the participants’ shape discrimination performance deteriorated significantly when the two objects on any given trial had to be compared simultaneously (one object explored with the left hand & a different object explored simultaneously with the right hand). A 2-way within-subjects analysis of variance (ANOVA) conducted upon the results shown in Fig. 6 revealed significant effects of both haptic exploration mode (simultaneous versus successive comparison, F(1, 4) = 11.0, p < 0.03; η^2^_p_ = 0.73) and surface curvature magnitude (F(2, 8) = 54.7, p < 0.0001; η^2^_p_ = 0.93). In addition, the haptic exploration mode × curvature magnitude interaction was significant (F(2, 8) = 4.6, p < 0.05, η^2^_p_ = 0.54). The increase in performance with increasing curvature magnitude for simultaneous stimulus comparison was quite linear. In contrast, in the successive comparison condition, the performance improvement obtained from an increase in stimulus curvature from 0.6 to 1.0 m^−1^ was smaller than that obtained from an increase in curvature from 0.2 to 0.6 m^−1^; this reduction was probably the result of a ceiling effect.

## General Discussion

Previous research by Kappers and Koenderink^17^ and Kappers *et al*.^18^ indicated that unimanual haptic exploration produced superior judgments of shape, at least for simple curved surfaces (cylindrical and quadric surfaces). These initial results were certainly interesting, because they suggested that shape performance was best if the required somatosensory cortical processing were limited to one cerebral hemisphere (haptic shape comparison of objects felt separately by the left and right hands requires interhemispheric communication across cerebral commissures and somatosensory processing in both cerebral hemispheres). One purpose of the current study was to determine whether this advantage for unimanual over bimanual exploration generalized to the perception and discrimination of more complex and ecologically valid objects. A second purpose of the current study was to determine whether somatosensory processing differs in younger and older adults; if there was a general perceptual deficit that accompanies bimanual haptic exploration, would this deficit increase with increasing age? One might expect such an outcome, given that past research^23–28^ has demonstrated that aging is associated with deterioration in interhemispheric communication.

The results of the current Experiment 1 for both younger and older participants (see Fig. 2) were quite different from the earlier studies of Kappers and colleagues^17,18^, suggesting that either the particular stimulus objects or some other methodological difference was responsible. Experiment 2 was conducted to determine whether the type of stimulus shape *per se* differentially affects unimanual and bimanual haptic performance; the combined results of Experiments 1 and 2 (Figs 2 and 5) clearly demonstrate that unimanual shape discrimination is not generally superior to bimanual shape discrimination, either for simple curved surfaces or complex solid objects.

Despite the outcomes of the current Experiments 1 and 2, it is nevertheless true that Kappers and Koenderink^17^ found a significant difference in performance between their unimanual and bimanual exploration conditions. An additional possibility, unexplored in Experiments 1 and 2, is a potential difference between the perceptual informativeness of successive and simultaneous comparison. In the current Experiments 1 and 2, the participants always haptically explored the stimulus objects sequentially; in the relevant studies of Kappers *et al*.^17,18^, the participants in the bimanual condition were allowed to explore the stimulus objects simultaneously with their left and right hands. To evaluate whether simultaneous haptic exploration is associated with a reduction in shape discrimination ability, we conducted Experiment 3. Indeed, the outcome of the current Experiment 3 was clear: while simultaneous haptic comparison of shape is possible (i.e., d’ values of 0.96 to 1.68 for curvature magnitudes of 0.6 m^−1^ or higher), it comes at a cost. Simultanous exploration does produce inferior shape discrimination performance when compared to successive haptic exploration (see Fig. 6). Why might this be the case? Our finding seems quite comparable to the classic studies involving dichotic listening^42,43^. Cherry^42^, for example, played auditory passages separately to participants’ left and right ears. While the participants could easily attend to and recognize speech presented to one ear, they could not effectively perceive auditory passages simultaneously from both ears. A comparison of the bimanual results of Experiments 2 (successive) and 3 (simultaneous) reveals a similar haptic phenomenon–if participants are only required to attend to one stimulus object at a time, its shape can be well recovered and discrimination performance will be high. If, however, participants are required to attend simultaneously to multiple stimulus objects, then there is a reduction in shape recovery and discrimination performance is reduced. This possible effect of divided attention upon the haptic discrimination of shape is reinforced by an analogous tactile finding by Craig^44^. In Craig’s study, tactile 2-D patterns were delivered simultaneously to his participants’ index and middle fingers. In one condition, participants were directed to attend to one particular finger; on each trial, they were required to identify one of 10 possible tactile patterns presented to that attended finger. In the divided attention condition, different tactile patterns were also presented to both index and middle fingers; *after* the stimuli for each trial were removed, however, the participants were cued by a visual display to identify either the pattern presented to the index finger or the pattern presented to the middle finger. Analogous to our results (Fig. 6), while Craig’s tactile patterns could be identified (much better than chance) when the two fingers’ stimuli were attended to simultaneously, there was a significant cost. The performance observed in the divided attention condition was much lower than that obtained during the directed attention condition (77 versus 93 percent correct, respectively).

Another possible explanation for our results (Fig. 6) beyond divided attention is interference between the hemispheres^1,45–48^. Our results for successive shape comparison demonstrate that the cerebral hemispheres can cooperate effectively under some conditions (i.e., high d’ values for successive comparison of objects whose attributes are initially processed in separate hemispheres). However, our results (Fig. 6) also demonstrate that human shape discriminability (as reflected by d′) is reduced by about 50 percent when haptic information about object shape must be compared simultaneously across the two hemispheres. It may be that while each hemisphere is engaged in processing the shape information coming from the contralateral hand during simultaneous exploration, it is less receptive to (or negatively affected by) the corresponding activity occurring in the opposite hemisphere. Thus, haptic shape discrimination performance is markedly reduced during simultaneous exploration as compared to the maximal performance obtained for successive exploration.

At this point in the discussion, it is very important to note that the simultaneous usage of two hands during haptic exploration does not in and of itself reduce shape discrimination ability. In a previous study by Crabtree and Norman^29^, participants used both hands to simultaneously explore/feel the same object shapes (bell peppers 1, 2, 3, 5, 7, 8, 11, and 12) as used in the current Experiment 1 (where the participants’ two hands, left & right, felt objects only successively). Both the prior experiment^29^ and the current Experiment 1 required the participants to perform the identical same/different shape discrimination task. The discriminabilities obtained in the two experiments (mean d′ = 1.942 for the experiment of Crabtree & Norman^29^; mean d′ = 2.007 for younger adults in the current Experiment 1) are essentially identical and are not significantly different (t(20) = 0.43, p = 0.67, 2-tailed). This similarity of performance demonstrates that the simultaneous use of two hands to feel objects (both hands were simultaneously used to feel each individual object)^29^ can be as effective as the separate/successive use of two hands (current Experiment 1) when discriminating 3-D object shape. What produces poor discrimination performance is the *simultaneous exploration of two different objects by two different hands* (current Experiment 3); thus, our overall results are consistent with either interhemispheric interference or divided attention.

As was pointed out earlier in this discussion, one of the purposes of the current study was to evaluate the potential adverse effects of aging upon bimanual shape discrimination; this potential effect (relatively good performance for older adults for unimanual haptic exploration, but reduced performance for bimanual haptic exploration) might occur as a consequence of the reduced interhemispheric communication that accompanies aging for other modalities and tasks^23–28^. Nevertheless, our current findings (see Fig. 2) are clear: not only is there no deterioration in shape discrimination performance accompanying bimanual haptic exploration in younger adults, there is also no decrement whatsoever for older adults. Because tactile excitatory input to the cerebral cortex initially arrives (e.g., in cortical area 3b) only from the contralateral hand, good performance for our bimanual shape discrimination condition requires effective interhemispheric communication. Our current results (white bars in Fig. 2) suggest that, at least for the sense of touch, increases in age do not necessarily result in a loss of effective functional communication between the two cerebral hemispheres.

Previous studies demonstrate that many types of information combine across the two hands^49–52^. Consider, for example, aftereffects of shape/curvature: feeling a convex surface with one hand (say the left) can produce a concave aftereffect for the other hand (right)^50–52^. This behavioral phenomenon obviously demonstrates that information from the two hands is combined at some locus within the somatosensory system. Obvious candidate locations would be cortical areas 2 and 5, which possess tactile neurons with bilateral receptive fields. Since this bilateral sensitivity depends upon effective callosal communication^15^, and our current results indicate preserved interhemispheric tactile communication in older adults, we therefore predict that curvature aftereffects across the hands should occur for older adults. Testing this possibility would be a logical choice for future research.

## Conclusion

Under conditions of successive (but not simultaneous) comparison, bimanual exploration produces haptic shape discrimination performance that can be as effective as that obtained from unimanual haptic exploration; for successive comparison, the two cerebral hemispheres of the brain are able to cooperate effectively to support the haptic discrimination of object shape.

## References

[1] Lappin JS, Foulke E (1973). Expanding the tactual field of view. Percept. Psychophys..

[2] Plaisier, M. A. & Ernst M. O. Two hands perceive better than one in *Haptics: Perception, Devices, Mobility, and Communication. EuroHaptics 2012. Lecture Notes in Computer Science, vol 7283*. (eds. Isokoski, P. & Springare, J.) 127–132 (Springer, 2012).

[3] Squeri V (2012). Two hands, one perception: How bimanual haptic information is combined by the brain. J. Neurophysiol..

[4] Wong JD, Wilson ET, Kistemaker DA, Gribble PL (2014). Bimanual proprioception: Are two hands better than one?. J. Neurophysiol..

[5] Talvas A, Marchal M, Lécuyer A (2014). A Survey on Bimanual Haptic Interaction. IEEE Trans. Haptics.

[6] Gardner, E. P. & Johnson, K. O. Touch in *Principles of neural science, 5th edition*. (eds. Kandel, E. R., Schwartz, J. H., Jessell, T. M., Siegelbaum, S. A., & Hudspeth, A. J.) 498–529 (McGraw Hill, 2013).

[7] Inui K, Wang X, Tamura Y, Kaneoke Y, Kakigi R (2004). Serial processing in the human somatosensory system. Cereb. Cortex.

[8] Iwamura Y (1998). Hierarchical somatosensory processing. Curr. Opin. Neurobiol..

[9] Kaas, J. H. Somatosensory system in The Human Nervous System, 2nd edition. (eds. Paxinos, G. & Mai, J. K.) 1059–1092 (Elsevier, 2004).

[10] Jones EG, Powell TPS (1969). Connexions of the somatic sensory cortex of the rhesus monkey: I. ipsilateral cortical connexions. Brain.

[11] Lipton ML, Fu KM, Branch CA, Schroeder CE (2006). Ipsilateral hand input to area 3b revealed by converging hemodynamic and electrophysiological analyses in macaque monkeys. J. Neurosci..

[12] Reed JL, Qi HX, Kaas JH (2011). Spatiotemporal properties of neuron response suppression in owl monkey primary somatosensory cortex when stimuli are presented to both hands. J. Neurosci..

[13] Sakata H, Takaoka Y, Kawarasaki A, Shibutani H (1973). Somatosensory properties of neurons in the superior parietal cortex (area 5) of the rhesus monkey. Brain Res..

[14] Taoka M, Toda T, Iwamura Y (1998). Representation of the midline trunk, bilateral arms, and shoulders in the monkey postcentral somatosensory cortex. Exp. Brain Res..

[15] Iwamura Y, Iriki A, Tanaka M (1994). Bilateral hand representation in the postcentral somatosensory cortex. Nature.

[16] Gazzaniga MS, Bogen JE, Sperry RW (1963). Laterality effects in somesthesis following cerebral commissurotomy in man. Neuropsychologia.

[17] Kappers AML, Koenderink JJ (1996). Haptic unilateral and bilateral discrimination of curved surfaces. Perception.

[18] Kappers AML, Koenderink JJ, te Pas SF (1994). Haptic discrimination of doubly curved surfaces. Perception.

[19] Nefs HT, Kappers AML, Koenderink JJ (2005). Intermanual and intramanual tactual grating discrimination. Exp. Brain Res..

[20] Fagot J, Lacreuse A, Vauclair J (1994). Hand-movement profiles in a tactual-tactual matching task: Effects of spatial factors and laterality. Percept. Psychophys..

[21] Norman JF (2012). Solid shape discrimination from vision and haptics: Natural objects (Capsicum annuum) and Gibson’s “feelies”. Exp. Brain Res..

[22] Norman JF, Bartholomew AN, Burton CL (2008). Aging preserves the ability to perceive 3-D object shape from static but not deforming boundary contours. Acta Psychol..

[23] Beaton, A. A., Hugdahl, K. & Ray, P. Lateral asymmetries and interhemispheric transfer in aging: A review and some new data in *Side Bias: A Neuropsychological Perspective* (eds Mandal, M. K., Bulman-Fleming, M. B., Tiwari, G.) 101–152 (Kluwer, 2000).

[24] Bellis TJ, Wilber LA (2001). Effects of aging and gender on interhemispheric function. J. Speech Lang. Hear. Res..

[25] Jeeves MA, Moes P (1996). Interhemispheric transfer time differences related to aging and gender. Neuropsychologia.

[26] Moes P, Jeeves MA, Cook K (1995). Bimanual coordination with aging: Implications for interhemispheric transfer. Dev. Neuropsychol..

[27] Sullivan EV, Pfefferbaum A (2006). Diffusion tensor imaging and aging. Neurosci. Biobehav. Rev..

[28] Voineskos AN (2012). Age-related decline in white matter tract integrity and cognitive performance: A DTI tractography and structural equation modeling study. Neurobiol. Aging.

[29] Crabtree CE, Norman JF (2014). Short-term visual deprivation, tactile acuity, and haptic solid shape discrimination. PLOS ONE.

[30] Norman JF, Bartholomew AN (2011). Blindness enhances tactile acuity and haptic 3-D shape discrimination. Atten. Percept. Psychophys..

[31] Norman JF, Norman HF, Clayton AM, Lianekhammy J, Zielke G (2004). The visual and haptic perception of natural object shape. Percept. Psychophys..

[32] Dellon, A. L. It’s academic but not functional in *Evaluation of sensibility and re-education of sensation in the hand* (ed. Dellon, A. L.) 95–113 (Williams & Wilkins, 1981).

[33] Cheeseman JR, Norman JF, Kappers AML (2016). Dynamic cutaneous information is sufficient for precise curvature discrimination. Sci. Rep..

[34] Desrosiers J, Hébert R, Bravo G, Dutil E (1996). Hand sensibility of healthy older people. J. Am. Geriatr. Soc..

[35] Moberg E (1958). Objective methods for determining the functional value of sensibility in the hand. J. Bone Joint Surg..

[36] Norman JF (2017). Aging and haptic-visual solid shape matching. Perception.

[37] Norman JF (2011). Aging and the haptic perception of 3D surface shape. Atten. Percept. Psychophys..

[38] Macmillan, N. A. & Creelman, C. D. *Detection theory: A user’s guide* (Cambridge University Press, 1991).

[39] Pont SC, Kappers AML, Koenderink JJ (1997). Haptic curvature discrimination at several regions of the hand. Percept. Psychophys..

[40] Norman JF (2013). Aging and curvature discrimination from static and dynamic touch. PLOS ONE.

[41] Pont SC, Kappers AML, Koenderink JJ (1999). Similar mechanisms underlie curvature comparison by static and dynamic touch. Percept. Psychophys..

[42] Cherry EC (1953). Some experiments on the recognition of speech, with one and with two ears. J. Acoust. Soc. Am..

[43] Moray N (1959). Attention in dichotic listening: Affective cues and the influence of instructions. Q. J. Exp. Psychol..

[44] Craig JC (1985). Tactile pattern perception and its perturbations. J. Acoust. Soc. Am..

[45] Palmer LM (2012). The cellular basis of GABA_B_-mediated interhemispheric inhibition. Science.

[46] Seyal M, Ro T, Rafal R (1995). Increased sensitivity to ipsilateral cutaneous stimuli following transcranial magnetic stimulation of the parietal lobe. Ann. Neurol..

[47] Forss N, Hietanen M, Salonen O, Hari R (1999). Modified activation of somatosensory cortical network in patients with right-hemisphere stroke. Brain.

[48] Chiarello C, Maxfield L (1996). Varieties of interhemispheric inhibition, or how to keep a good hemisphere down. Brain Cogn..

[49] Dupin L, Hayward V, Wexler M (2015). Direct coupling of haptic signals between hands. Proc. Natl. Acad. Sci. USA.

[50] Denisova K, Kibbe MM, Cholewiak SA, Kim S-H (2014). Intra- and intermanual curvature aftereffect can be obtained via tool-touch. *IEEE Trans*. Haptics.

[51] van der Horst BJ (2008). Intramanual and intermanual transfer of the curvature aftereffect. Exp. Brain Res..

[52] van der Horst BJ, Willebrands WP, Kappers AML (2008). Transfer of the curvature aftereffect in dynamic touch. Neuropsychologia.

